# Modulation of adipose tissue lipolysis and body weight by high-density lipoproteins in mice

**DOI:** 10.1038/nutd.2014.4

**Published:** 2014-02-24

**Authors:** H Wei, M M Averill, T S McMillen, F Dastvan, P Mitra, S Subramanian, C Tang, A Chait, R C LeBoeuf

**Affiliations:** 1 Division of Metabolism, Endocrinology and Nutrition, Department of Medicine, and Diabetes and Obesity Center of Excellence, University of Washington, Seattle, WA, USA; 2 Center of Cardiovascular Biology, Department of Medicine, University of Washington, Seattle, WA, USA

**Keywords:** HDL, caloric restriction, mice, obesity, hormone-sensitive lipase

## Abstract

**Background:**

Obesity is associated with reduced levels of circulating high-density lipoproteins (HDLs) and its major protein, apolipoprotein (apo) A-I. As a result of the role of HDL and apoA-I in cellular lipid transport, low HDL and apoA-I may contribute directly to establishing or maintaining the obese condition.

**Methods:**

To test this, male C57BL/6 wild-type (WT), apoA-I deficient (apoA-I^−/−^) and apoA-I transgenic (apoA-I^tg/tg^) mice were fed obesogenic diets (ODs) and monitored for several clinical parameters. We also performed cell culture studies.

**Results:**

ApoA-I^−/−^ mice gained significantly more body weight and body fat than WT mice over 20 weeks despite their reduced food intake. During a caloric restriction regime imposed on OD-fed mice, apoA-I deficiency significantly inhibited the loss of body fat as compared with WT mice. Reduced body fat loss with caloric restriction in apoA-I^−/−^ mice was associated with blunted stimulated adipose tissue lipolysis as verified by decreased levels of phosphorylated hormone-sensitive lipase (p-HSL) and lipolytic enzyme mRNA. In contrast to apoA-I^−/−^ mice, apoA-I^tg/tg^ mice gained relatively less weight than WT mice, consistent with other reports. ApoA-I^tg/tg^ mice showed increased adipose tissue lipolysis, verified by increased levels of p-HSL and lipolytic enzyme mRNA. In cell culture studies, HDL and apoA-I specifically increased catecholamine-induced lipolysis possibly through modulating the adipocyte plasma membrane cholesterol content.

**Conclusions:**

Thus, apoA-I and HDL contribute to modulating body fat content by controlling the extent of lipolysis. ApoA-I and HDL are key components of lipid metabolism in adipose tissue and constitute new therapeutic targets in obesity.

## Introduction

Obesity is a major health problem in the United States^1, 2, 3^ and is associated with increased susceptibility to various diseases, particularly type 2 diabetes mellitus and cardiovascular diseases.^4, 5, 6^ Obesity is also associated with dyslipidemia including elevations of very low-density lipoprotein triglyceride (TG) and reduced concentrations of high-density lipoprotein (HDL) cholesterol.^7, 8, 9^ Reduced levels of HDL cholesterol and its primary apolipoprotein (apo), apoA-I, are also inversely correlated to the risk for cardiovascular disease.^10^ Mechanisms by which HDL and apoA-I provide cardiovascular protection are reverse cholesterol transport,^11, 12^ suppression of inflammation,^13, 14, 15^ immune activation,^14, 15^ oxidation^15^ and inhibition of coagulation and platelet activation.^16^ Most of these processes involve alterations in cellular cholesterol homeostasis.

Adipose tissue contains a significant amount of whole-body cholesterol stores,^17, 18, 19^ the levels of which must be tightly maintained.^20^ As HDL and apoA-I have key roles in tissue cholesterol removal and because TG and cholesterol homeostasis are coupled in adipocytes,^19, 20, 21, 22^ we wondered whether levels of circulating HDL and apoA-I could participate in modulating body weight and composition. There is some evidence of this from animal studies. Reduced fat mass and improved insulin sensitivity were observed for mice overexpressing apoA-I (apoA-I transgenic, apoA-I^tg/tg^)^23^ and for mice treated with apoA-I mimetics.^23, 24^ In apoA-I deficient (apoA-I^−/−^) mice as compared with wild-type (WT) controls, fat mass was increased and glucose tolerance reduced.^25^ Metabolic pathways affected by HDL/apoA-I levels were attributed to alterations in energy expenditure, anti-oxidation enzyme levels and 5′-adenosine monophosphate-activated protein kinase phosphorylation.^25^ However, detailed molecular pathways by which HDL/apoA-I drive metabolic pathways have not been established.

Toward this end, we used apoA-I^−/−^ and apoA-I^tg/tg^ mice to investigate whether alterations in lipolysis could have a role in the HDL/apoA-I regulation of body weight. Cell culture studies were also used to test mechanisms by which HDL and apoA-I alter lipolysis. Our hypothesis is that as a consequence of reduced levels of circulating HDL and apoA-I in obesity, there is a reduction in cholesterol efflux and thereby lipolysis leading to retention of adipocyte TG.

Our data indicate that apoA-I possesses an anti-obesity property, which is consistent with previous studies.^23, 24^ HDL and apoA-I modulate body weight through control of adipocyte lipolysis via hormone-sensitive lipase phosphorylation (p-HSL). Our finding that apoA-I deficiency results in a diminished ability to lose body weight and fat content with caloric restriction support these results. Using 3T3-L1 adipocytes, we further show that HDL and apoA-I modulate adipocyte lipolysis by directly promoting catecholamine-elicited lipolysis but not basal lipolysis. Overall, our findings suggest that HDL and apoA-I have a direct role in the regulation of body weight and are potential pharmacological targets for the treatment of obesity.

## Materials and methods

### Materials

3T3-L1 cells were purchased from Zen-Bio (Research Triangle Park, NC, USA). Dulbecco's modified Eagle's medium and other culture reagents were purchased from Thermo Scientific (Rockford, IL, USA). Fatty acid-free bovine serum albumin was purchased from Roche Molecular Biochemicals (Indianapolis, IN, USA). Isobutyl methyl xanthine, dexamethasone, insulin, isoproterenol (ISOP) and 8-Bromo-cyclic adenosine monophosphate (cAMP) were purchased from Sigma (St Louis, MO, USA). Human HDL was obtained as a gift from Dr Jay Heinecke (Department of Medicine, University of Washington, Seattle, WA, USA), and the method of HDL preparation has been published previously.^26^ Human apoA-I was purified as previously described.^27^

### Mice

Male apoA-I^−/−^, apoA-I^tg/tg^ and C57BL/6J WT mice were purchased from the Jackson Laboratory (Bar Harbor, ME, USA; #002055, #001927 and #000664, respectively). Animals were housed in pathogen-free conditions, in a temperature and humidity-controlled environment (12-h light/dark cycle). Two *in vivo* experiments were performed. First, WT and apoA-I^−/−^ mice (8 weeks old) fed with an obesogenic diet (OD) without added cholesterol (34.9% fat, 26.3% carbohydrate; D12492, Research Diets, Inc., New Brunswick, NJ, USA) for 20 weeks. These mice were then subjected to a 2-week caloric restriction regimen. During caloric restriction, mice were individually housed and given 60% of their *ad libitum* food consumption. At the end of the experiment, mice were fasted for 12 h before retro-orbital blood collection and killing. A second experiment was performed using apoA-I^tg/tg^ and WT mice (12 weeks old) fed an OD that contains cholesterol (35.5% fat, 36.6% carbohydrate with 0.15% added cholesterol, No.F4997, Bio-Serv, Frenchtown, NJ, USA) for 24 weeks. At the end of the experiment, mice were fasted for 4 h and bled before killing. The apoA-I^tg/tg^ and corresponding WT mice are the same mice used previously to characterize adipose tissue inflammation in response to high-fat diet feeding.^14^ For all mice, body weight was measured weekly and daily food intake was determined during the last 4 weeks of feeding the diets. For both experiments, plasma was stored at −80 °C before further analyses. For the first experiment, epididymal and inguinal adipose tissue, and liver were collected, weighed and snap-frozen in liquid nitrogen and stored at −80 °C before further analysis. All animal procedures were reviewed and approved by the Institute for Laboratory Animal Research Guide for Care and Use of Laboratory Animals and the University of Washington Animal Use Committee.

### Body composition

Analyses of lean and fat mass content were done using quantitative magnetic resonance (EchoMRI 3-in-1 machine whole-body composition analyzer; Echo MRI, LLC., Houston, TX, USA).^28, 29^ For the apoA-I^−/−^ study, body composition was performed before and 10 days after caloric restriction was initiated. Percentage of fat mass lost was calculated as: X%=(loss of fat (g)/original fat mass (g)) × 100.

### Analytical protocols

Plasma total cholesterol and TG levels were determined using colorimetric kits as described previously.^30^ HDL cholesterol was quantified by measuring total cholesterol in the supernatant from polyethylene glycol precipitated serum. Plasma leptin levels were determined as described previously.^30^

### Real-time quantitative reverse transcriptase-PCR

Total RNA samples were isolated from whole epididymal adipose tissue using TRIzol Reagent from Invitrogen (Carlsbad, CA, USA) and quantified by spectrometry. First-strand complementary DNAs were synthesized using Superscript III reverse transcriptase (EPICENTRE Biotechnologies, Madison, WI, USA) and random hexamer primers. Messenger RNA levels of ATP-binding cassette A1 (ABCA1), ABCG1, ApoE, low-density lipoprotein receptor, perilipin-1 (PLIN1), PLIN2, adipose tissue lipase, HSL, PPARalpha, PGC-1alpha, CPT1, UCP2 and L32 were measured by real-time reverse transcriptase-PCR using 7500 real-time PCR System (Applied Biosystems, Foster City, CA, USA) and SYBR Green Master Mix (Bioline USA Inc., Tauton, MA, USA). PCR primers were designed using Primer Bank (http://pga.mgh.harvard.edu/primerbank/) (Supplementary Table 1). PCR reactions were performed in duplicate, and the data were analyzed with the delta delta cycle threshold method.^21, 30^ After normalization to internal control L32 standard, the results for each target gene were expressed as fold change of WT control.

### 3T3-L1 adipocyte differentiation and lipolysis

3T3-L1 preadipocytes were grown and differentiated by standard techniques.^14^ Adipocytes were then maintained in Dulbecco's modified Eagle's medium/10% fetal bovine serum by changing medium every 2 days. For lipolysis experiments, 3T3-L1 adipocytes at 11 days post-differentiation were pre-incubated with phenol red and serum-free Dulbecco's modified Eagle's medium containing 0.2% free fatty acid-free bovine serum albumin overnight to remove the impact of lipoproteins contained in 10% serum. Cells were incubated with human HDL, human apoA-I, ISOP or 8-Br-cAMP or mixtures of these components for 4 h. Conditioned medium was harvested and glycerol levels quantified as an indicator for lipolysis using the Free Glycerol Determination Kit (Sigma) according to the manufacturer's instructions.

### Immunoblotting

Cell lysate was prepared from 3T3-L1 adipocytes or mouse epididymal white adipose tissue. Equal amounts of protein (20 μg) were electrophoresed on 4–12% sodium dodecyl sulfate polyacrylamide gels and then electro-transferred to a ProTran nitrocellulose membrane (Schleicher & Schuell, Riviera Beach, FL, USA). Blots were incubated with the rabbit anti-mouse p-HSL (Cell Signaling Technology, Danvers, MA, USA), rabbit anti-mouse HSL (Cell Signaling Technology) and rabbit anti-mouse glyceraldehyde 3-phosphate dehydrogenase primary antibody (Abcam, Boston, MA, USA), and then probed with horseradish peroxidase-conjugated goat anti-rabbit secondary antibody (Chemicon International, Temecula, CA, USA). Blots were developed using SuperSignal pico ECL kit (Pierce, Thermo Scientific, Rockford, IL, USA). Molecular band intensity was determined by densitometry using NIH ImageJ software (developed at the U.S. National Intitutes of Health and available on the Internet at http://rsb.info.nih.gov/nih-image/).

### Statistical analysis

Data are reported as mean±s.e.m. and statistical significance was established at *P*<0.05. Student's *t*-test and non-parametric analysis of variance (Bonferroni correction) analyses followed by multiple comparisons were performed as appropriate, focusing on genotype and diet status. Dose dependency was analyzed by linear regression. Growth curves were analyzed by repeated-measures analysis of variance using R programming (http://ww2.coastal.edu/kingw/statistics/R-tutorials/repeated.html).

## Results

### ApoA-I deficiency increased adiposity, reduced food intake and inhibited weight loss with caloric restriction

**Figure 1:**
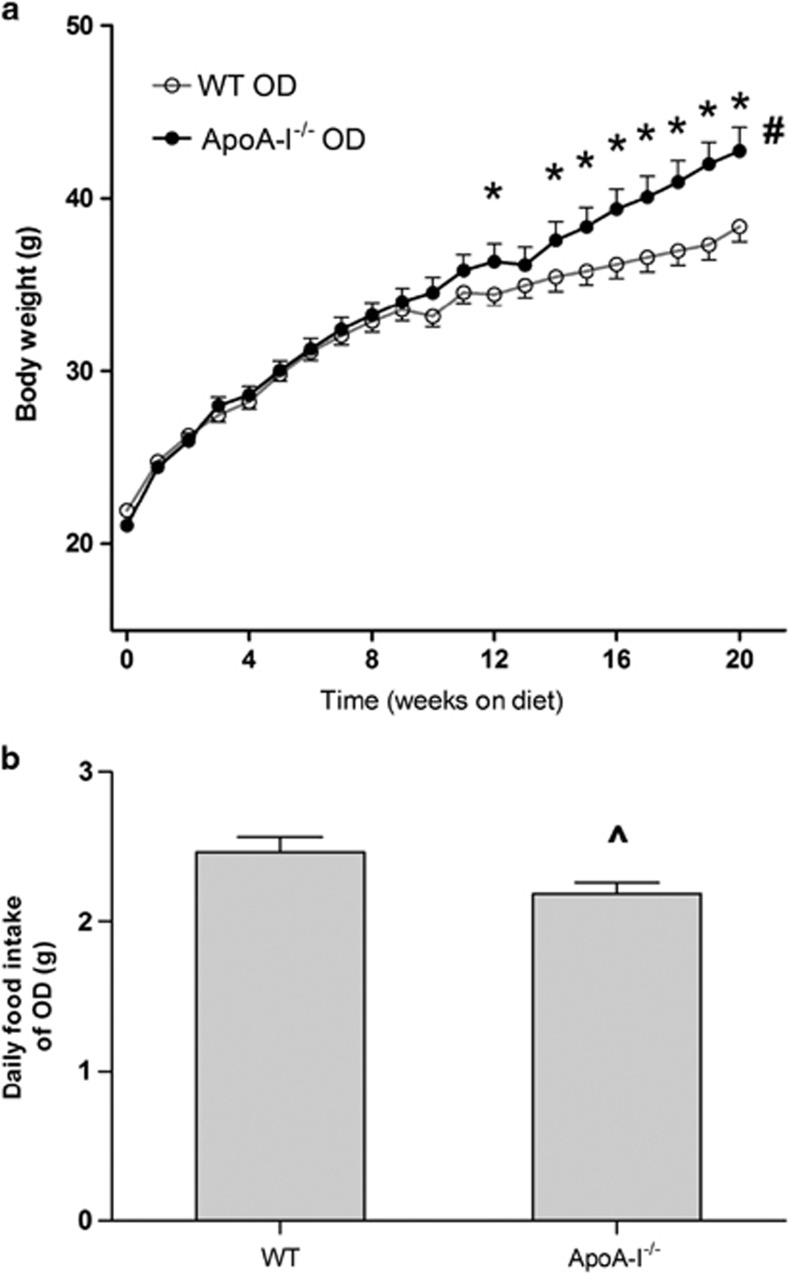
ApoA-I^−/−^ mice show increased body weight gain compared with WT mice with OD feeding. Male WT and apoA-I^−/−^ mice were fed the OD for 20 weeks beginning at 8 weeks of age. Body weights and daily food intake were recorded as described in Materials and Methods section. (**a**) Body weights of WT and apoA-I^−/−^ mice over 20 weeks of OD feeding. The asterisks denote statistically significant differences in body weight between apoA-I^−/−^ and WT mice at particular time points (*P*<0.05, *t*-test). ‘^#^' denotes statistically significant differences between genotypes for overall growth curves between apoA-I^−/−^ and WT mice (*P*<0.0005, repeated-measures analysis of variance). (**b**) Daily food intake of WT and apoA-I^−/−^ mice. Data are presented as mean±s.e.m. (*n*=17). The ‘^' denotes statistically significant differences in food intake between apoA-I^−/−^ and WT mice (*P*<0.05, *t*-test).

To study potential roles of HDL and apoA-I in obesity, we first compared body weights of apoA-I^−/−^ and WT mice fed OD for 20 weeks. At 8 weeks of age, apoA-I^−/−^ and WT mice of comparable body weights (21 g) were fed an OD without added cholesterol for 20 weeks. Absolute body weight was significantly increased for apoA-I^−/−^ mice starting at 12 weeks of feeding, resulting in an increase of 16% body weight gain for apoA-I^−/−^ over that for WT mice by 20 weeks (Figure 1a). At 20 weeks of OD feeding, apoA-I^−/−^ mice had significantly increased fat mass as compared with WT mice (43.3±1.0% for apoA-I^−/−^; 36.2±2.1% for WT; *P*<0.001, *n*=16). Interestingly, we observed a statistically significant reduction in daily food intake by 11% for apoA-I^−/−^ mice as compared with WT mice (Figure 1b). We also confirmed that plasma HDL cholesterol levels were substantially decreased in apoA-I^−/−^ mice as compared with WTs and this is consistent with a remarkable reduction in plasma total cholesterol levels in apoA-I^−/−^ mice (Table 1). These data are comparable with lipid levels seen by others for this mouse strain.^31^

**Figure 2:**
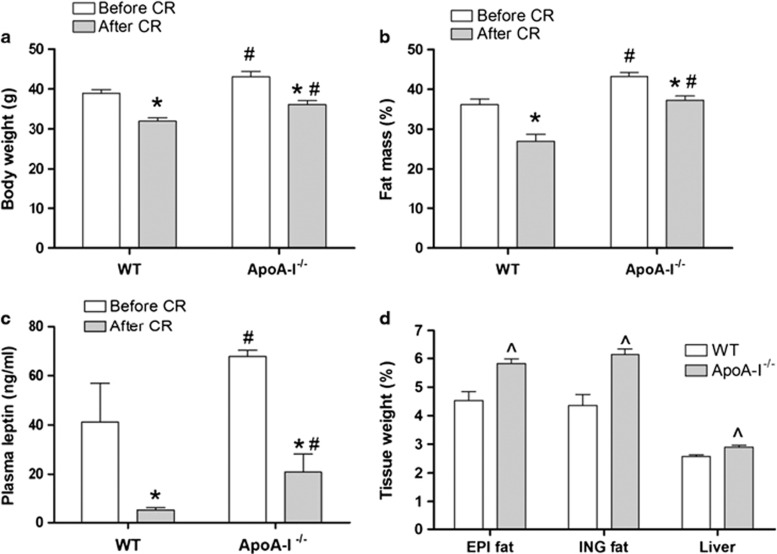
ApoA-I^−/−^ mice lose less weight and fat mass during caloric restriction compared with WT mice. Male WT and apoA-I^−/−^ mice were fed the OD for 20 weeks beginning at 8 weeks of age. Then mice were individually housed and subjected to a caloric restriction regimen by providing mice with 60% of their normal food intake for 2 weeks. Body compositions of mice were assessed by quantitative magnetic resonance at 2 days before and 10 days after caloric restriction. Epididymal (EPI) fat, inguinal (ING) fat and liver were collected and weighed; plasma leptin levels were measured as described in Materials and methods section. (**a**) Body weight of WT and apoA-I^−/−^ mice before (white) and after (gray) caloric restriction. (**b**) Percentage fat mass of WT and apoA-I^−/−^ mice before (white) and after (gray) caloric restriction. (**c**) Plasma leptin levels of WT and apoA-I^−/−^ mice before (white) and after (gray) caloric restriction. (**d**) Tissue weight of WT (white) and apoA-I^−/−^ (gray) mice as percentage of body weight after caloric restriction. Data are presented as mean±s.e.m. (*n*=17 for **a**, **b** and **d** and *n*=4 for **c**). The asterisk denotes statistically significant differences before and after caloric restriction in the same strain (*P*<0.05, analysis of variance (ANOVA)). ‘^#^' Denotes statistically significant differences between genotypes before or after caloric restriction (*P*<0.05, ANOVA). ‘^' Denotes statistically significant differences in tissue weights between WT and apoA-I^−/−^ mice (*P*<0.05, *t*-test).

These data are consistent with apoA-I's previously described role in modulating body weight and fat with OD feeding.^23, 24, 25^ Thus, we expected that body weight loss for mice under caloric restriction would be inhibited in the absence of apoA-I. To test this, we subjected mice were fed the OD for 20 weeks to caloric restriction for 2 weeks. Although both genotypes lost body weight (Figure 2a) and relative fat mass (Figure 2b) with caloric restriction, the amount of total mass and relative fat lost was greater for WT than apoA-I^−/−^ mice. The relative loss in body weight was 18.0±0.6% for WT as compared with 16.0±0.6% for apoA-I^−/−^ mice (*P*<0.05, *t*-test). In terms of relative body fat, apoA-I^−/−^ remained fatter than WT mice, losing 27.6±1.0% fat as compared with 39.9±2.0% for WT (*P*<0.05, *t*-test). Relative loss of lean body mass was not significantly different between the genotypes (5.86±0.56 for WT and 6.77±0.37 for apoA-I^−/−^, *n*=8–9). The higher fat mass for apoA-I^−/−^ in pre- and post-calorie restriction states was reflected in the levels of circulating leptin that were higher for apoA-I^−/−^ than WT mice in each of the fed and caloric restriction groups (Figure 2c).

Further analysis of body weight and fat mass content of apoA-I^−/−^ and WT mice showed that both caloric restriction and genotype are highly significant contributors to differences in body weight and fat mass (*P*<0.05, analysis of variance). Following caloric restriction, apoA-I^−/−^ mice also had greater weights of major fat storage tissues, including epididymal fat, inguinal fat and liver (by 28%, 42% and 12%, respectively) as compared with WT mice (Figure 2d).

### ApoA-I deficiency inhibited adipose tissue lipolysis

**Figure 3:**
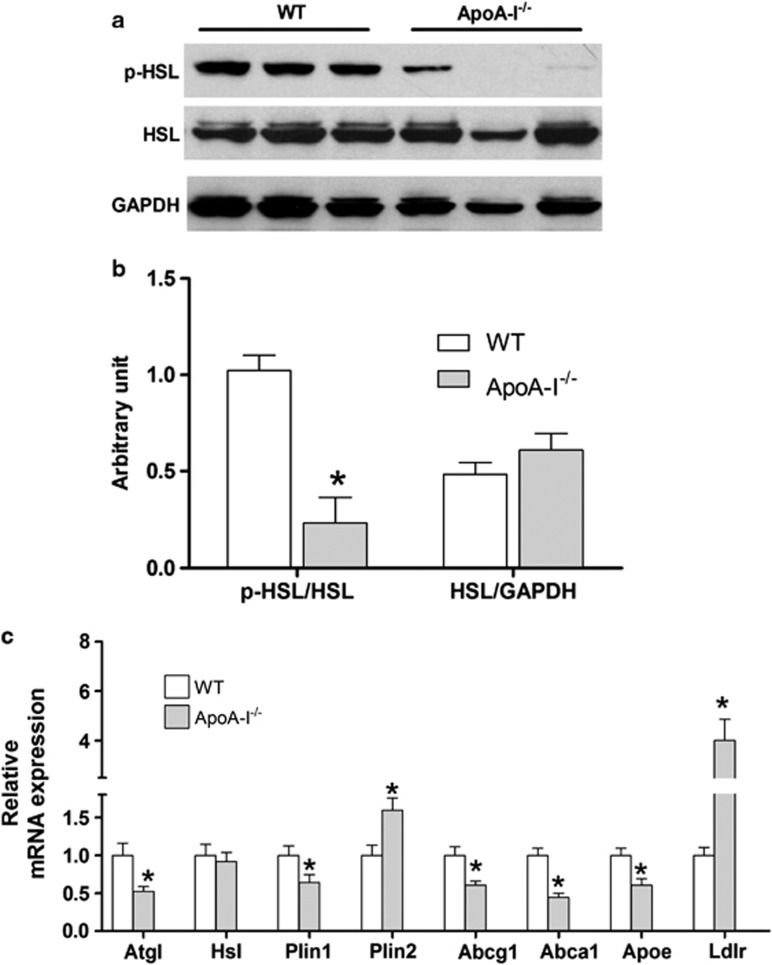
ApoA-I^−/−^ mice have lower expression of adipose tissue lipase (ATGL), decreased p-HSL and differentially expressed lipid metabolism genes in white adipose tissue (WAT) compared with WT mice during caloric restriction. Animals were fed the OD and subjected to caloric restriction as described in the legend of Figure 2. WAT mRNA levels of lipase and lipid metabolism genes were quantified by reverse transcriptase-PCR, and cell lysates were immunoblotted for p-HSL, HSL and glyceraldehyde 3-phosphate dehydrogenase (GAPDH) as described in Materials and Methods section. (**a**) Immunoblots for p-HSL, HSL and GAPDH in WAT of WT and apoA-I^−/−^ mice. Data presented are from three representative animals in each group. (**b**) Densitometric data for p-HSL (calculated by pHSL/HSL) and HSL (calculated by HSL/GAPDH). Data are presented as mean±s.e.m. (*n*=5). (**c**) mRNA levels of lipase and lipid metabolism genes in WAT of WT (white bar) and apoA-I^−/−^ (gray bar) mice. Data are presented as fold change from WT mice as mean±s.e.m. (*n*=17). The asterisk denotes statistically significant differences from WT mice (*P*<0.05, *t*-test).

One mechanism by which apoA-I and HDL may modulate body weight is via central regulation of appetite, as suggested by our findings with food consumption. Another mechanism is via modulation of adipocyte lipolysis. To determine whether the reduced ability to lose body fat with caloric restriction was associated with reduced adipose tissue lipolytic activity, we quantified levels of protein and expression for several lipolytic enzymes (Figure 3). Full hydrolysis of TG depends on the activity of three key enzymes, adipose tissue lipase, HSL and monoacylglycerol lipase.^32^ The hallmark for HSL activity is its activation by phosphorylation.^32, 33^ ApoA-I^−/−^ mice had significantly reduced (by 77%) p-HSL levels than WT mice (Figures 3a and b). HSL protein levels were comparable between genotypes as were the HSL mRNA expression levels. Adipose tissue lipase mRNA levels were markedly reduced (by 48%) for the apoA-I^−/−^ strain, supporting a state of reduced stimulated lipolysis in adipose tissue of apoA-I^−/−^ versus WT mice (Figure 3c).

Consistent with increased fat mass, the mRNA level of Plin2, a major protein constituent of the globule surface of lipid droplets regulating lipid storage,^34, 35, 36^ increased significantly in apoA-I^−/−^ as compared with WT mice. On the other hand, Plin1, a lipid droplet protein required for stimulated lipolysis,^37, 38^ showed a significant reduction in mRNA level in apoA-I^−/−^ mouse adipose tissue. Expression levels of the low-density lipoprotein receptor were increased by 1.6-fold indicating upregulated cellular cholesterol intake, whereas genes responsible for cellular cholesterol efflux, such as ABCG1, ABCA1 and apoE were markedly decreased (56%, 39% and 40%, respectively) in apoA-I^−/−^ as compared with WT mice (Figure 3c). Together with lipolysis enzymes, mRNA expression levels support a pattern of lipid retention by attenuated lipolytic activity in adipose tissue from apoA-I^−/−^ over that seen for WT mice.

### Adipose tissue lipolysis was greater for apoA-I^tg/tg^ mice

ApoA-I^tg/tg^ mice are known to be leaner than WTs following the feeding of a high-fat diet.^23^ Thus, we hypothesized that apoA-I^tg/tg^ mice would show increased white adipose tissue lipolysis. To test this hypothesis, we had the opportunity to utilized adipose tissues already available which were taken from apoA-I^tg/tg^ and WT mice under fasting conditions used in another study.^14^ Our apoA-I^tg/tg^ mice exhibited significantly higher plasma total cholesterol levels than WT mice (by 2.3-fold) due to marked augmentation of HDL cholesterol levels (Table 1) as seen previously.^39^

**Figure 4:**
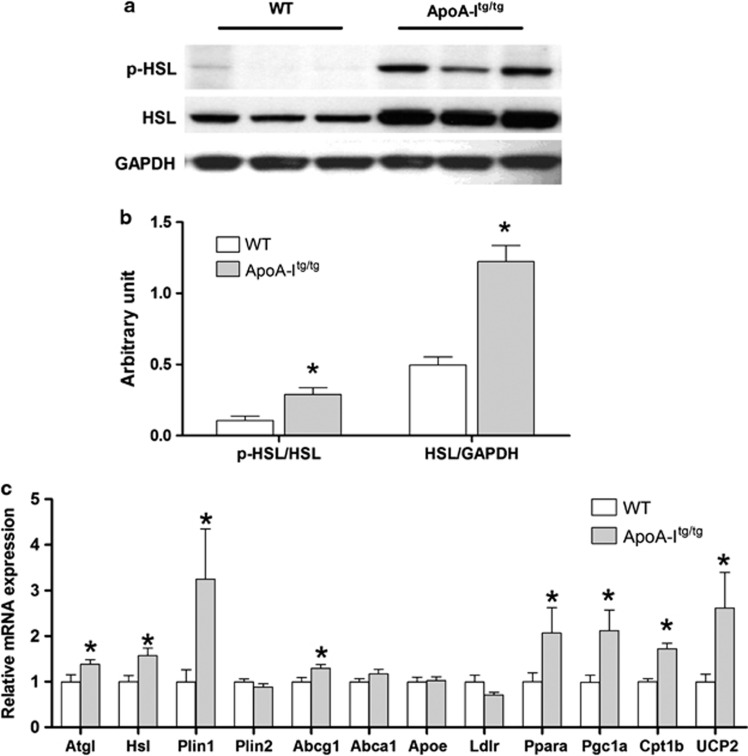
ApoA-I^tg/tg^ mice possess higher epididymal white adipose tissue (WAT) expressions of adipose tissue lipase (ATGL) and HSL as well as greater p-HSL compared with WT mice. Animals were fed the OD as described in the legend of Figure 1. Total RNA and cell lysates were prepared from epididymal WAT. mRNA levels of lipase and lipid metabolism genes were quantified by reverse transcriptase-PCR and normalized to L32 levels; cell lysate were immunoblotted for p-HSL, HSL and glyceraldehyde 3-phosphate dehydrogenase (GAPDH) as described in Materials and Methods section. (**a**) Immunoblots of p-HSL, HSL and GAPDH in WAT of WT and apoA-I^tg/tg^ mice. Data presented are from three representative animals in each group. (**b**) Densitometric data of p-HSL (calculated by pHSL/HSL) and HSL (calculated by HSL/GAPDH). Data are presented as mean±s.e.m. (*n*=5). (**c**) mRNA levels of lipase, lipid metabolism and fatty acid oxidation genes in WAT of WT (white bar) and apoA-I^tg/tg^ (gray bar) mice. Data are presented as fold change from values of WT mice as mean±s.e.m. (*n*=11). The asterisk denotes statistically significant differences from WT mice (*P*<0.05, *t*-test).

Although different diets were used among our and previous studies,^23^ and consistent with reports of greater leanness in apoA- I^tg/tg^ mice,^23^ we found that apoA-I^tg/tg^ mice had a 2.4-fold increase in white adipose tissue HSL protein levels as well as a 2.7-fold elevation in p-HSL as compared with WT controls (Figures 4a and b). Expression levels for HSL and adipose tissue lipase were significantly upregulated in apoA-I^tg/tg^ mice by 38% and 57%, respectively, as compared with WT mice (Figure 4c). ApoA-I^tg/tg^ mice also showed a significant threefold increase in Plin1 mRNA level compared with WT mice. In addition, we also observed significantly augmented mRNA levels of several genes involved in fatty acid oxidation (Pparα, PGC1α and CPT1β) and energy expenditure (UCP2) for apoA-I^tg/tg^ mice, reflecting higher TG hydrolysis and free fatty acid oxidation in their adipose tissue (Figure 4c). Finally, mRNA levels for ABCG1 were significantly increased for apoA-I^tg/tg^ mice. Overall, expression levels support a pattern of elevated lipolysis with active cholesterol mobilization from adipose tissue of apoA-I^tg/tg^ mice as compared with WTs.

### HDL and apoA-I promote catecholamine-elicited lipolysis but not basal lipolysis in 3T3-L1 adipocytes

To determine whether HDL and apoA-I directly influence adipocyte lipolysis, we evaluated basal and stimulated lipolysis by quantification of glycerol release and p-HSL levels from cultured 3T3-L1 cells incubated with HDL or apoA-I.

**Figure 5:**
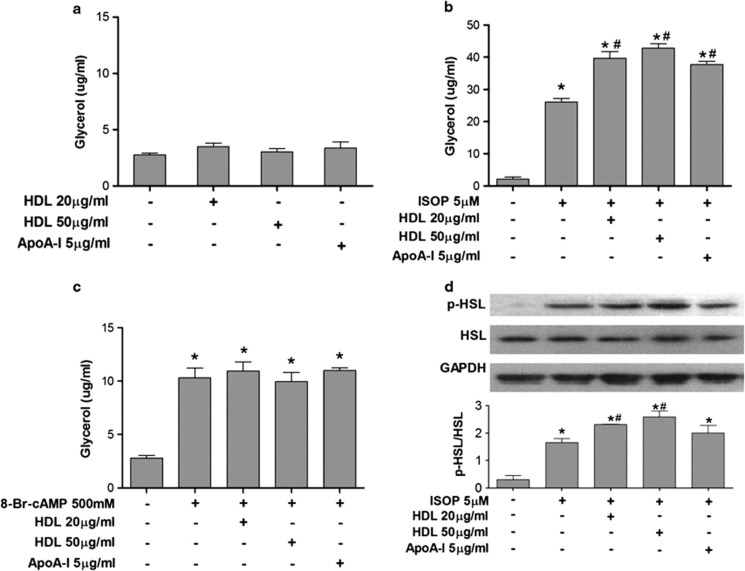
HDL promotes catecholamine but not cAMP-stimulated lipolysis in 3T3-L1 adipocytes. 3T3-L1 adipocytes at day 10 after differentiation were cultured in Dulbecco's modified Eagle's medium containing fatty acid-free 0.2% bovine serum albumin for 24 h, and followed by incubation with or without lipolytic stimuli, 5 μM ISOP or 500 mM 8-Br-cAMP, and with or without human HDL and apoA-I in indicated concentrations for 4 h. Net release of glycerol into conditioned media was measured as the indicator of lipolysis. Whole-cell lysates were prepared and immunoblotted for p-HSL, HSL and glyceraldehyde 3-phosphate dehydrogenase (GAPDH) as described in Materials and methods section. (**a**) Basal lipolysis of 3T3-L1 adipocytes with HDL and apoA-I. (**b**) Catecholamine ISOP-stimulated lipolysis with HDL and apoA1. (**c**) cAMP-stimulated lipolysis with HDL and apoA-I. (**d**) Immunoblots of p-HSL, HSL and GAPDH in 3T3-L1 adipocytes under ISOP stimulation with HDL and apoA-I (upper panel). Densitometric data for p-HSL/HSL (lower panel). The image is one representative immunoblot from three independent experiments. The asterisk denotes statistically significant differences compared with control (analysis of variance (ANOVA), *P*<0.05). ‘^#^' Denotes statistically significant differences compared with cells treated with 5 μM ISOP (ANOVA, *P*<0.05).

Incubation of 3T3-L1 adipocytes with HDL or apoA-I did not alter basal lipolysis (Figure 5a). In contrast, co-treatment of cells with HDL (20 or 50 μg ml^–1^) or apoA-I (5 μg ml^–1^) significantly enhance ISOP-elicited lipolysis by 45% (Figure 5b).

It has been established that stimulation of the β-adrenergic receptor by ISOP leads to production of intracellular cAMP as a second messenger for triggering lipolysis. We further tested if HDL can similarly promote cAMP-stimulated lipolysis in 3T3-L1 adipocytes. Co-treatment of cells with 8-Br-cAMP 500 mM and HDL (20 or 50 μg ml^–1^) or apoA-I (5 μg ml^–1^) showed no effect on glycerol release as compared with 8-Br-cAMP alone (Figure 5c).

We then measured the extent of p-HSL in cultured adipocytes to further assess the lipolytic potential of these cells. In the absence of ISOP, p-HSL was nearly undetectable. ISOP treatment caused a significant increase of p-HSL, and co-treatment with HDL or apoA-I resulted in a further augmentation of p-HSL (Figure 5d).

Overall, our data support the concept that apoA-I and HDL modulate the extent of TG lipolysis in adipocytes during weight gain and weight loss conditions and that this is mediated at the level of cell surface receptor systems.

## Discussion

In this study, we sought to determine whether HDL and apoA-I have a direct role in the regulation of body weight and to identify mechanisms by which apoA-I and HDL alter adipose tissue lipid metabolism. This goal was initiated by noting that metabolic syndrome is associated with obesity, insulin resistance and an atherogenic dyslipidemia that includes reduced levels of circulating HDL. Also, others have observed that altering circulating apoA-I levels in mice result in changes in body fat mass.^23, 25^ Here, we confirm and extend these studies, and demonstrate that a major mechanism by which apoA-I and HDL modulate body fat is by driving adipocyte lipolysis. ApoA-I contributes to regulating the loss of body fat during caloric restriction, a regimen often used for the treatment of obesity.

Treatment of 3T3-L1 cells with HDL or apoA-I resulted in significantly enhanced lipolysis. This enhancement was not seen for basal lipolysis but for ISOP-stimulated (demand) lipolysis. Consistent with the cell culture results, adipose tissue lipolysis was stimulated for fasted apoA-I^tg/tg^ and apoA-I^−/−^ mice, as evidenced by direct measures of p-HSL as well as changes in the gene expression profiles for lipases and lipid transport proteins. In addition, we show for the first time that apoA-I deficiency leads to a reduced ability to lose body fat under a caloric restriction regime. These data support the idea that apoA-I is a key regulator of adipose tissue lipid homeostasis.

Of note are the changes in mRNA levels for apoE and *Plin* genes in adipose tissue taken from mice with different levels of apoA-I. ApoE expression in adipose tissue of calorically restricted apoA-I^−/−^ mice was significantly reduced and this is in concert with elegant studies demonstrating a key role of apoE as a nonsecreted protein controlling adipocyte lipid uptake.^40^ Plins are a family of proteins with a variety of functions that coat lipid droplets in adipocytes. Plin2 is associated with the globule surface of lipid droplets and has a role in regulating lipid storage properties,^36^ whereas Plin1 functions as a scaffold, both suppressing basal and facilitating stimulated lipolysis.^37, 38^ Stimulation of the beta-2-adrenergic receptor by catecholamine activates adenylate cyclase, producing intracellular cAMP as a second messenger. This is followed by downstream activation of cAMP-dependent protein kinase that in turn, activates lipolysis via the phosphorylation of Plin1 and recruitment of HSL, the rate-limiting enzyme in TG hydrolysis, to the lipid droplet.^32, 41^ The coordinated decreases of pHSL and Plin1 in apoA-I^−/−^ mice suggest that apoA-I deficiency leads to an orchestrated inhibition of adipose lipolytic machinery. This is reinforced by the observation that mice overexpressing apoA-I had increased levels of Plin1 mRNA and pHSL in adipose tissue.

Adipocytes contain about 25% of total body cholesterol most of which is free cholesterol located primarily (∼70%) in the monolayer membrane of the lipid droplet.^17, 18, 19^ During lipolysis, the lipid droplet shrinks in size^21, 42^ leaving excess membrane components and raising local concentrations of free cholesterol. As adipocytes cannot degrade cholesterol, this toxic lipid must be removed from the adipocyte. How free cholesterol is trafficked from the lipid droplet to the plasma membrane is not completely understood although unidirectional diffusion along a gradient through the endoplasmic reticulum to the plasma membrane surface has been suggested.^22^ In addition, lipid trafficking proteins such as caveolins and cavins may participate in clearing the lipid droplet of free cholesterol.^43, 44^

Demand lipolysis as seen during fasting and weight loss regimes results in a decrease of plasma membrane cholesterol concentration^45^ and cholesterol depletion leads to an increase in beta-2-adrenergic receptor-stimulated cAMP production.^46, 47^ Adipocytes are able to undergo receptor-mediated cholesterol efflux.^45, 48, 49^ Thus, the ability of HDL and apoA-I to reduce the plasma membrane cholesterol concentration may contribute to efficient lipolysis. Interestingly, when we used a cAMP derivative as the stimulus for lipolysis in our cell culture studies, HDL and apoA-I conveyed no lipolysis enhancing effect, suggesting that the effects of HDL and apoA-I occurred up-stream of cAMP. We postulate that adrenergic receptor-mediated signaling may be the target of HDL and apoA-I regulation. In fact, 3T3-L1 cells treated with apoA-I or HDL showed markedly reduced plasma membrane cholesterol concentrations.^14^ Also, the lipid composition and organization of the lipid droplet membrane influences the locations and activities of several key proteins required for lipolysis including Plin and HSL.^50^ Thus, maintaining a cholesterol efflux ‘gradient' during lipolysis may allow adipocytes to rapidly adapt to energy demand by coupling TG hydrolysis to healthy maintenance of lipid droplet cholesterol content.

In conclusion, our *in vitro* and *in vivo* data point out that HDL and apoA-I modulate adipose tissue lipid utilization by directly promoting catecholamine-elicited but not basal lipolysis possibly through a receptor-mediated mechanism. Low HDL/apoA-I levels associated with obesity contribute to an inability to lose fat effectively via a negative influence on adipose lipolysis, and hence causing a vicious circle of fat accumulation. Further research addressing the effectiveness of elevating apoA-I protein or HDL particle levels to correct defective lipolytic machinery of obese subjects is an area of clinical importance. Our findings may offer a new therapeutic target for treating obesity and provide a greater understanding of the metabolic changes and biological adaptations occurring in adipose tissue during weight gain and loss.

## Figures and Tables

**Table 1:** Plasma lipid levels for apoA-I^tg/tg^ and apoA-I^−/−^ mice fed the OD

*Trait*	*WT*	*ApoA-I*^−/−^	*WT*	*ApoA-I*^*tg/tg*^
Total cholesterol (mg dl^–1^)	146±5	61±5^a^	314±20	726±23^a^
HDL cholesterol (mg dl^–1^)	100±3	31±2^a^	189±3	311±5^a^
Triglyceride (mg dl^–1^)	27±1	14±1^a^	54±3	110±8^a^

Abbreviations: apo, apolipoprotein; apoA-I^−/−^, apoA-I deficient; apoA-I^tg/tg^, apoA-I transgenic; HDL, high-density lipoprotein; OD, obesogenic diet; WT, wild type.

WT and apoA-I^−/−^ mice (*n*=17 each genotype) were fed OD diet without cholesterol for 20 weeks. WT and apoA-I^tg/tg^ mice (*n*=11 each genotype) were fed OD diet containing cholesterol for 24 weeks. Data are presented as mean±s.e.m.

a *P*<0.05 between genotypes.
