# Supplementary material for: Modulation of adipose tissue lipolysis and body weight by high-density lipoproteins in mice
Source: Nutr Diabetes. 2014 Feb 24;4(2):e108–. doi: 10.1038/nutd.2014.4 (PMC3940828; doi:10.1038/nutd.2014.4)
Supplement: Supplementary Table 1 [file nutd20144x1.doc]

**Supplemental TABLE. List of PCR primers. Sequences are given as 5’ ** 3’.

| Abca1 | Forward: | AACTCTACATCTCCCTTCCCG |
| --- | --- | --- |
|  | Reverse: | CTCCTGTCGCATGTCACTCC |
| Abcg1 | Forward: | GTGGATGAGGTTGAGACAGACC |
|  | Reverse: | CCTCGGGTACAGAGTAGGAAAG |
| Apoe | Forward: | CTGACAGGATGCCTAGCCG |
|  | Reverse: | CGCAGGTAATCCCAGAAGC |
| Atgl | Forward: | TGTGGCCTCATTCCTCCTAC |
|  | Reverse: | TCGTGGATGTTGGTGGAGCT |
| Cd68 | Forward: | acttcgggccatgtttctct |
|  | Reverse: | ggctggtaggttgattgtcgt |
| Chi3l3 | Forward: | CAGGTCTGGCAATTCTTCTGAA |
|  | Reverse: | GTCTTGCTCATGTGTGTAAGTGA |
| Cpt1b | Forward: | TGGGACTGGTCGATTGCATC |
|  | Reverse: | CAGGGTTTGTCGGAAGAAGAAAA |
| Hsl | Forward: | GCTGGGCTGTCAAGCACTGT |
|  | Reverse: | GTAACTGGGTAGGCTGCCAT |
| IL6 | Forward: | TAGTCCTTCCTACCCCAATTTCC |
|  | Reverse: | TTGGTCCTTAGCCACTCCTTC |
| Ldlr | Forward: | TGACTCAGACGAACAAGGCTG |
|  | Reverse: | ATCTAGGCAATCTCGGTCTCC |
| L32 | Forward: | TTAAGCGAAACTGGCGGAAAC |
|  | Reverse: | TTGTTGCTCCCATAACCGATG |
| Nos2 | Forward: | GTTCTCAGCCCAACAATACAAGA |
|  | Reverse: | GTGGACGGGTCGATGTCAC |
| Pgc1a | Forward: | TATGGAGTGACATAGAGTGTGCT |
|  | Reverse: | GTCGCTACACCACTTCAATCC |
| Plin2 | Forward: | GACCTTGTGTCCTCCGCTTAT |
|  | Reverse: | ACGGGTACTGATCCTTTGTACT |
| Ppara | Forward: | AGAGCCCCATCTGTCCTCTC |
|  | Reverse: | ACTGGTAGTCTGCAAAACCAAA |
